# Supplementary material for: Utilizing Longitudinal Chest X-Rays and Reports to Pre-Fill Radiology Reports
Source: ArXiv. 2023 Jun 14:arXiv:2306.08749v1. Preprint. [Version 1] (PMC10370215)
Supplement: Supplementary file 1 [file NIHPP2306.08749v1-supplement-1.pdf]

Supplementary Material

Hierarchical Memory-Driven Encoder

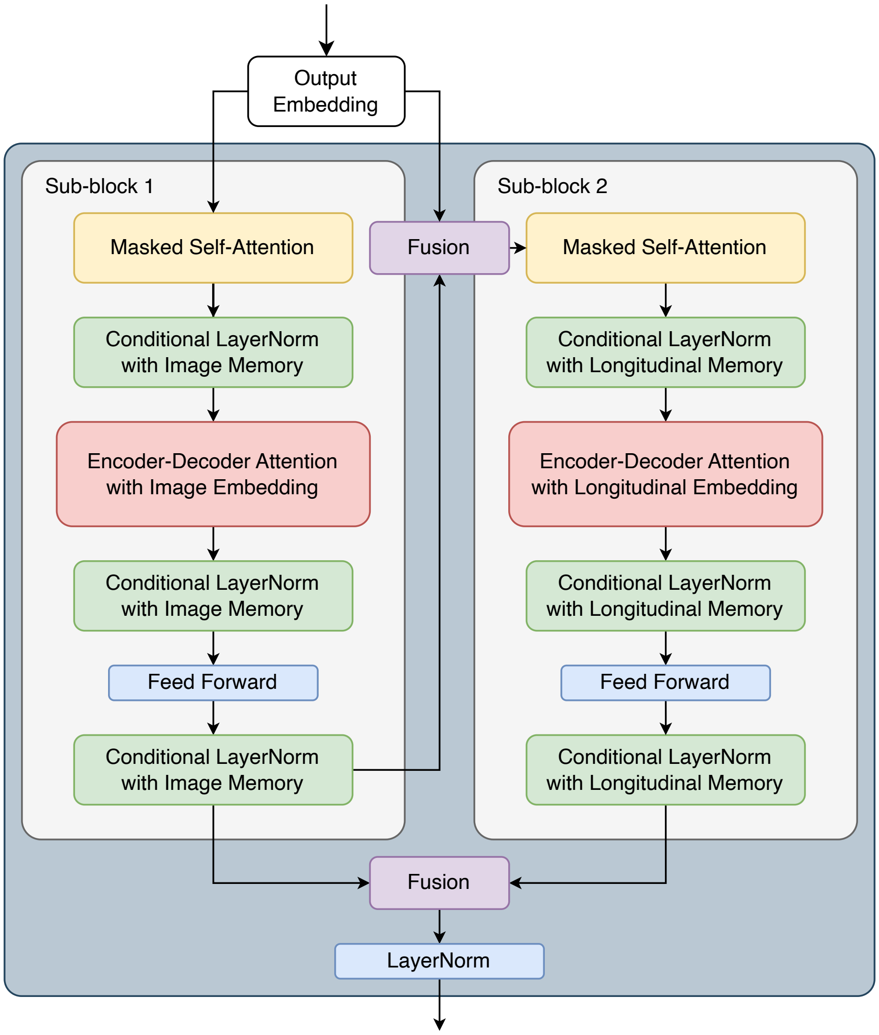

Fig. 3: The sub-components of the hierarchical memory-driven decoder.

Implementation Details

Table 3: Implementation Details

**Component**	**Details**
Pre-trained Model	ResNet-101
Feature Extraction	2048 dimensions for each feature set from ImageNet
Backbone Structure	Transformer with 3 layers, 8 attention heads, 512 hidden states dimensions
Image Encoders	Same transformer-based encoder for current and previous images
Text Encoder	Separate transformer encoder without shared parameters
Initialization	Random initialization for transformers
Relational Memory	512 dimensions, 8 attention heads, 3 memory slots
Loss Function	Cross-entropy loss at word level
Training Epochs	30 epochs
Optimizer	ADAM optimizer
Learning Rate	5e-5 for visual extractor, 1e-4 for other parameters
Learning Rate Decay	0.8 per epoch

One more case

Previous Image

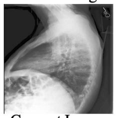

Previous Report

ap and lateral views of chest demonstrate a right upper lobe consolidation with some areas of air bronchogram. background multifocal opacities with volume loss and chronic scarring are unchanged. there is no large pleural effusion. cardiac size is normal.

Current Image

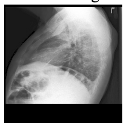

Current Report (Ground Truth)

pa and lateral chest radiographs were obtained. a right upper lobe consolidation with air bronchograms is similar to . focal tubular lucency within the opacity is new and may reflect cavitation dilated airways or spared lung parenchyma. opacity in the right lower lobe has progressed since the prior study. there is no effusion or pneumothorax. cardiac and mediastinal contours are normal. there is mild thickening of the left major fissure.

Labels:[x 0 x x 1 x 1 -1 x 0 0 x x x]

Baseline-Generated Current Report

pa and lateral views of the chest provided. there is no focal consolidation effusion or pneumothorax. the cardiomediastinal silhouette is normal. imaged osseous structures are intact. no free air below the right hemidiaphragm is seen.

Labels:[1 0 x x x x 0 x x 0 0 x x x]

Ours-Generated Current Report

pa and lateral views of the chest were provided . there is extensive consolidation and increased opacity in the right upper lobe concerning for pneumonia. there is no pleural effusion or pneumothorax . the cardiomediastinal silhouette is normal. imaged osseous structures are intact. no free air below the right hemidiaphragm is seen.

Labels:[x 0 x x 1 x 1 x x 0 0 x x x]

Fig. 4: An example of pre-filled “findings” section of a report.
